# Index to Predict Waiting Times for Pediatric Liver Transplantation

**DOI:** 10.1111/petr.70313

**Published:** 2026-04-01

**Authors:** Eric Shin, Ashley Montgomery, Megan Crawford, Garrett Wortham, Chase Robinson, John Goss, Nhu Thao Nguyen Galván, Abbas Rana

**Affiliations:** ^1^ Department of Student Affairs Baylor College of Medicine Houston Texas USA; ^2^ Division of Abdominal Transplantation, Michael E. DeBakey Department of Surgery Baylor College of Medicine Houston Texas USA

## Abstract

**Background:**

Hundreds of pediatric candidates are added to the liver transplant waitlist annually, yet wait times remain uncertain. This study identifies the variables associated with changes in wait time and creates a predictive points score to predict the likelihood of receiving a deceased‐donor pediatric liver transplant within 1 year of listing.

**Methods:**

A retrospective analysis of 5246 pediatric liver transplant candidates (2014–2024) was conducted using de‐identified OPTN/UNOS data. Variables including demographics, lab values, and medical history were analyzed using univariate and multivariate logistic regression. Significant predictors from the multivariate model were used to develop a weighted points system, with model performance assessed via ROC analysis.

**Results:**

19 variables were statistically significant. The 3 significant variables most positively associated with receiving a transplant are: high volume transplant center (Odds Ratio [OR]: 3.79, *p*‐value [*p*] < 0.001), Pacific Islander race (OR: 2.96, *p* = 0.049), and medium volume transplant center (OR: 2.53, *p* < 0.003). The 3 significant variables most negatively associated with receiving a transplant are: serum sodium > 150 mEq/L (OR: 0.37, *p* = 0.002), acute hepatic necrosis (OR: 0.53, *p* < 0.001), and bilirubin 1–2 mg/dL (OR: 0.68, *p* < 0.001). The model demonstrated moderate predictive accuracy (c‐statistic = 0.66).

**Conclusion:**

This study identifies key predictors of pediatric liver transplant wait time and introduces a predictive scoring system to improve clinical decision‐making. This tool provides a more accurate framework for identifying vulnerable candidates and can assist clinicians in pursuing technical variant or living donor options for those at highest risk of prolonged waitlist times.

## Graphical abstract

Using national OPTN/UNOS data, this study identifies clinical and transplant center factors that influence pediatric liver transplant wait times and introduces a points‐based model to estimate the likelihood of transplantation within 1 year. The model highlights candidates at risk for prolonged waiting and supports earlier consideration of alternative transplant strategies.

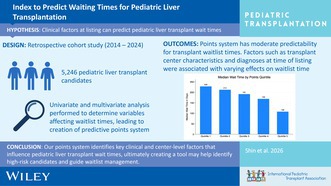

**Abbreviations**
AC: acuity circle
CI: confidence interval
MELD: model for end‐stage liver disease
OPTN: organ procurement and transplantation network
PELD: pediatric end‐stage liver disease
ROC: receiver operating characteristic
SOFT: survival outcomes following liver transplantation
TV: technical variant
UNOS: united network for organ sharing

## 1 Introduction

Demand for pediatric liver allografts remains high despite recent efforts to prioritize pediatric candidates. The Organ Procurement and Transplant Network (OPTN), a federal system under the United Network for Organ Sharing (UNOS), enacted a novel plan in 2020 to prioritize pediatric candidates for pediatric donors, a strategy long recommended by clinicians [1, 2]. However, pretransplant mortality remains high, with an overall rate at 5.6% and even reaching 21.7% in candidates under 1 year according to a 2021 report [2]. Studies have shown increased wait times may be associated with long‐term deficits and poor intellectual and academic outcomes [3]. Furthermore, the emotional impact of the waitlist is considerable, with studies showing candidates experiencing higher anxiety, depression, and PTSD compared to controls [4, 5]. A major driver of stress is the inherent uncertainty of the waitlist, as the lack of information on the factors that influence wait times contributes to the families' uncertainty and emotional strain.

Current literature in pediatric liver transplantation focuses on post‐transplant survival and long‐term outcomes. These studies, often spanning decades, gather data on the health outcomes of transplant patients such as liver failure and rejection as well as risk factors impacting survival such as socioeconomic and lifestyle factors [1, 6, 7, 8]. Analysis of candidates' pre‐transplant experiences is comparatively lacking. Though OPTN notes a decrease in wait times from 2006 to 2017, they do not analyze variables that significantly affect these times [1]. Clinicians and patients would benefit from a study that includes data from recent years along with a broader analysis of variables impacting wait times.

This novel study aims to fill the gap by analyzing and discovering statistically significant variables associated with changes in waitlist times in pediatric liver transplantation. A broad range of variables including demographic factors, pre‐transplant diagnoses, and laboratory values were examined, as well as transplant center volume and utilization of deceased technical variant (TV) livers. TV grafts, which include living donor and deceased donor split/partial grafts, are alternatives to traditional whole liver transplants that impact waitlist times and outcomes [9]. By uncovering these variables, we hope to reduce the uncertainty surrounding the waitlist process for patients and their families, while also providing clinicians data to create more individualized care plans for candidates on the waitlist.

## 2 Methods

### 2.1 Study Population

We performed a retrospective analysis with data from the United Network for Organ Sharing (UNOS) database from 2014 to 2024. All data were de‐identified prior to analysis. Patients were excluded if they were aged 18 or older, had a previous organ transplant, were listed for multiple transplants, received a non‐deceased donor liver transplant, or received a transplant prior to January 1st, 2014. Living donor liver transplantation was excluded from the development of the points system because its availability is driven by elective, candidate‐specific donor identification rather than the systemic, biological, and geographic factors that govern deceased‐donor organ allocation. 5246 candidates met all the criteria and were thus analyzed in this study.

### 2.2 Statistical Analysis

Analysis was performed using StataBE 19 (Stata Corp, College Station, TX). Univariate and multivariate logistic regression analyses were performed to determine which variables were significantly correlated (*p* < 0.05) with receiving a transplant within 1 year of listing. Only variables found to be significant in the univariate analysis were included in the multivariate analysis. A points system was created based on the multivariate analysis. The model's ability to predict wait times was validated using ROC analysis. The outcome of interest for this analysis was defined as transplantation within 1 year. Therefore, a higher odds ratio (OR) indicates an increased probability of transplant within 1 year of listing, and conversely a lower OR is correlated with a decreased probability of transplant.

The predictive model was stratified into quintiles and graphed by the median days on the waitlist. A Kaplan–Meier (KM) curve was also created with the quintiles.

### 2.3 Missing Data

Variables with < 100% observations underwent five iterations of imputation via predictive matching with fifth nearest neighbor discriminant analysis. Variables that underwent imputation are BM and initial serum creatinine. Several variables had a 5245/5246 entry completion, and using discretion, these variables were not imputed.

### 2.4 Variables

Only variables recorded at the time of listing from the OPTN/UNOS database were considered in the study. Continuous variables were categorized by clinical relevance. For instance, the separation of BMI into categories was accomplished using guidelines outlined by the World Health Organization [10]. Reference ranges were determined using clinical judgment and prior studies [11].

Because the decision to receive whole liver versus technical variants is unknown at the time of listing, the type of graft received was not incorporated into the points system. Instead, listing transplant center characteristics are known at listing, and these variables were incorporated into the system. Center‐level characteristics were analyzed by stratifying the 88 transplant centers into two categories: volume and expertise. To assess volume, centers were divided into tertiles based on total transplant count. To assess surgical expertise, centers were categorized by their use of deceased technical variants (TV) based on proportion of transplants that were TV. Centers with zero TV utilization formed the first tier, while the remaining active TV‐utilizers were split between moderate and high TV utilization.

The following groups of variables were evaluated in the univariate analysis: age, blood group, body mass index (BMI), race/ethnicity, insurance type (private vs. public), ascites, status 1A/1B listing, encephalopathy, dialysis status, international normalized ratio (INR), bilirubin, serum albumin, serum creatinine, serum sodium, acute hepatic necrosis, non‐cholestatic cirrhosis, cholestatic cirrhosis, biliary atresia, metabolic disorder, malignancy, transplant center volume, and transplant center TV utilization.

The following variables were tested in the multivariate analysis: age: < 1, 5–10, 10–14, and 14–18; blood group: A, B, and AB; BMI: < 18 and 25–30; weight (kg): < 10, 40–50, and > 50; race: Pacific Islander, African American; insurance type: public; status 1A/B; ascites; serum bilirubin 1–2; dialysis; serum creatinine: > 2; serum sodium: < 130, 130–135, 145–150, and > 150; diagnoses: acute hepatic necrosis, non‐cholestatic cirrhosis, biliary atresia, metabolic disorder, malignancy; transplant center volume; transplant center TV utilization.

### 2.5 Predictive Model

Variables found to be significant in the multivariate analysis were used to create the points system. This system was based on Rana et al.'s SOFT score [12]. Each significant variable was assigned a point proportional to its respective odds ratio (OR); an OR of 0.78 would yield a score of −22 and an OR of 2.21 would yield a score of 121, for instance. The sum of the points of each present variable yields the points score. The ability of this points system to predict transplant within 1 year of listing was validated with ROC analyses.

## 3 Results

### 3.1 Demographics

The study cohort is composed of 5246 pediatric liver candidates, listed between January 1st, 2014 to 2024. The full demographics table is found in Table 1. The largest age group was infants (≤ 1 year) at 45%, followed by late adolescents (14–17) at 14%. The most common weight was < 10 kg at 39%, followed by 10–20 kg at 22% and > 50 kg at 18%. The majority of candidates (65%) were classified as underweight with BMI < 18, followed by normal BMI (18–25) at 27%. Less than 10% of candidates were listed as overweight or obese.

**TABLE 1:** Demographics table.

Variable	Entry completion (%)	Number of people	Percent	Median days on WL	Standard deviation
*Age* (*years*)					
≤ 1	100	2384	45.44%	63	303
> 1 and ≤ 2		363	6.92%	57	383
> 2 and ≤ 5		520	9.91%	50.5	368
> 5 and ≤ 10		618	11.78%	61	396
> 10 and ≤ 14		633	12.07%	57	402
> 14 and < 18		728	13.88%	63.5	448
*Weight* (*kg*)	100				
0–10		2056	39.19%	63	294
10–20		1173	22.36%	56	363
20–30		473	9.02%	63	394
30–40		302	5.76%	66	398
40–50		308	5.87%	66	365
> 50		934	17.80%	51.5	455
*MELD/PELD Score*	99.99				
≥ 20 and < 24		383	7.30%	46	261
≥ 24 and < 28		344	6.56%	20.5	214
≥ 28 and < 32		275	5.24%	14	95
≥ 32 and < 36		182	3.47%	6.5	116
≥ 36 and < 40		126	2.40%	5.5	60
≥ 40 and < 44		96	1.83%	4	24
≥ 44 and < 46		29	0.55%	9	17
≥ 46 and < 50		41	0.78%	3	131
≥ 50		46	0.88%	5.5	58
*Blood group*	100				
A		1704	32.48%	50	351
B		702	13.38%	63	358
O		2649	50.50%	69	380
AB		182	3.47%	49	204
*BMI‐for‐age* (*kg/m* ^*2*^)	99.65				
< 18		3434	65.46%	64	349
> 18 and ≤ 25		1427	27.20%	57	379
> 25 and ≤ 30		263	5.01%	42	352
> 30 and ≤ 35		85	1.62%	32	567
> 35 and ≤ 40		26	0.50%	44	614
> 40		11	0.21%	7	156
*Status 1A/B*		1143	21.79%	8	118.47
*Race/ethnicity*	100				
White		2460	46.89%	60	372
African American		856	16.32%	60.5	283
Hispanic		1307	24.91%	68	367
Asian		373	7.11%	56	433
Native American		50	0.95%	37	276
Pacific Islander		31	0.59%	32	281
Multiracial		138	2.63%	55.5	479
*Insurance type*	99.47				
Private		2062	39.31%	55.5	407
Public		2932	55.89%	67	337
*Ascites*	99.99	2623	50.00%	55	332
*Encephalopathy*	99.99	2249	42.87%	41	307
*Dialysis*	100	125	2.38%	5	213
*INR*	99.99				
< 0.8		2	0.04%	290.5	373
> 0.8 and ≤ 1.1		1822	34.73%	94	447
> 1.1		3422	65.23%	46	304
*Creatinine* (*mg/dL*)	96.32				
< 1.5		5157	98.30%	62	365
> 1.5 and ≤ 2.0		63	1.20%	4	189
> 2.0		26	0.50%	3	108
*Sodium* (*mEq/L*)	88.74				
< 130		158	3.01%	33.5	132
≥ 130 and ≤ 135		1040	19.82%	54	287
> 135 and ≤ 145		4250	81.01%	67	381
> 145 and ≤ 150		161	3.07%	11	393
> 150		50	0.95%	6	142
*Albumin* (*g/dL*)	99.99				
≤ 2.0		134	2.55%	25.5	92
> 2.0 and ≤ 2.5		605	11.53%	33	279
> 2.5 and ≤ 3.0		1163	22.17%	49	304
> 3.0		3344	63.74%		
*Bilirubin* (*mg/dL*)	99.99				
≤ 1.0		1540	29.36%	72.5	412
> 1.0 and ≤ 2.0		518	9.87%	116.5	465
> 2.0		3188	60.77%	49	180
*Diagnosis*					
Acute hepatic necrosis		705	13.44%	6	275
Non‐cholestatic cirrhosis		326	6.21%	87.5	477
Cholestatic cirrhosis		239	4.56%	88	316
Biliary atresia		1783	33.99%	97	429
Metabolic disorder		737	14.05%	83	332
Malignancy		493	9.40%	28	109
Graft failure		1	0.02%	54	N/A

*Note:* All variables analyzed in the study are listed with corresponding median waitlist length.

Candidates identifying as white were the most common race (47%), followed by Hispanic (25%), African American (16%), Asian (7%), multiracial (3%), Native American (0.95%), and Pacific Islander (0.59%). The most prevalent blood type was O (51%), followed by type A (32%), type B (13%), and AB (3%).

The lower MELD/PELD scores were more prevalent with 7.3% having a score ≤ 20, 6.6% at 20–24, and subsequently higher scores decreasing in prevalence. 50% of candidates had ascites, 43% had encephalopathy, and 2.38% were on dialysis. For lab values, the majority (98%) of candidates had a creatinine ≤ 1.5 mg/dL, 81% had a sodium 135–145 mEq/L, 64% had an albumin > 3 g/dL, 65% had an INR > 1.1, and 55% had a bilirubin > 2.

The most common diagnosis of candidates at listing was biliary atresia (34%), followed by acute hepatic necrosis (13%), metabolic disorder (14%), malignancy (9.4%), non‐cholestatic cirrhosis (6%), and cholestatic cirrhosis (5%).

There were 88 transplant centers at listing. The low, medium, and high tertiles have median yearly transplants of 1, 28, and 124 transplants respectively. Centers with zero TV utilization had a TV to total transplant ratio of 0, medium TV centers had a ratio of 0.03, and high TV centers had a ratio of 0.14. Reference Tables S1 and S2 for center characteristics.

### 3.2 Univariate and Multivariate Analysis

A univariate analysis was performed on all variables listed in Table 1. Among these, 33 variables were found to be statistically significant in association with wait times. A full list can be found in Table 2. These 33 variables were included in a multivariate regression analysis. This multivariate analysis revealed 19 variables that were correlated with changes in waitlist times at a statistically significant level.

**TABLE 2:** Significant Variables in Univariate Analysis.

Variable	Odds ratio	*p*	95% CI
*Age* (*years*)			
≤ 1	1.28	< 0.001	1.14–1.44
> 2 and ≤ 5	*Reference*		
> 5 and ≤ 10	1.23	0.029	1.02–1.48
> 10 and ≤ 14	0.83	0.04	0.70–0.99
> 14 and ≤ 18	0.54	< 0.001	0.46–0.63
*Blood group*			
A	1.22	0.002	1.07–1.38
B	1.2	0.043	1.01–1.43
AB	2.12	< 0.001	1.45–3.11
O	*Reference*		
*BMI* (*kg/m* ^*2*^)			
≤ 18	1.15	0.029	1.01–1.29
> 18 and ≤ 25	*Reference*		
> 25 and ≤ 30	0.7	0.006	0.54–0.90
*Weight (kg)*			
≤ 10	1.3	< 0.001	1.16–1.47
> 20 and ≤ 30	*Reference*		
> 40 and ≤ 50	0.66	< 0.001	0.52–0.83
≥ 50	0.62	< 0.001	0.54–0.72
*Race/ethnicity*			
White	*Reference*		
African American	1.19	0.032	1.02–1.40
Pacific Islander	3.12	0.034	1.09–8.93
*Insurance type*			
Public	1.17	0.01	1.04–1.31
*Clinical parameters*			
Ascites	1.32	< 0.001	1.17–1.48
Dialysis	0.64	0.015	0.45–0.92
*Bilirubin* (*mg/dL*)			
≤ 1.0	*Reference*		
> 1.0 and ≤ 2.0	0.61	< 0.001	0.50–0.73
*Sodium* (*mEq/L*)			
≤ 130	1.7	0.007	1.16–2.50
> 130 and ≤ 135	1.27	0.002	1.09–1.48
> 135 and ≤ 145	*Reference*		
> 145 and ≤ 150	0.62	0.003	0.45–0.85
> 150	0.28	< 0.001	0.16–0.49
*Creatinine* (*mg/dL*)			
≤ 1.5	*Reference*		
> 2.0	0.41	0.001	0.25–0.68
*Status 1A/B*	0.82	0.005	0.71–0.94
*Diagnosis*			
Acute hepatic necrosis	0.41	< 0.001	0.35–0.48
Non‐cholestatic cirrhosis	0.64	< 0.001	0.51–0.80
Biliary atresia	1.21	0.002	1.07–1.38
Metabolic disorder	2.03	< 0.001	1.68–2.46
Malignancy	2.51	< 0.001	1.97–3.21
*Center volume*			
Low tertile	*Reference*		
Medium tertile	0.7	< 0.001	0.61–0.82
High tertile	1.57	< 0.001	1.37–1.81
*Center TV Rate*			
Low TV ulitization	0.48	0.023	0.26–0.90
Medium TV utilization	0.36	0.011	0.16–0.79
High TV utiliation	*Reference*		

*Note:* The odds ratio, *p*‐value, and 95% confidence interval are listed along with the reference variable.

19 variables were statistically significant in the multivariate analysis as listed in Table 3. The 3 significant variables most positively associated with receiving a transplant are: high volume transplant center (Odds Ratio [OR]: 3.79, *p*‐value [*p*] < 0.001), Pacific Islander race (OR: 2.96, *p* = 0.049), and medium volume transplant center (OR: 2.53, *p* < 0.003). The 3 significant variables most negatively associated with receiving a transplant are: serum sodium > 150 mEq/L (OR: 0.37, *p* = 0.002), acute hepatic necrosis (OR: 0.53, *p* < 0.001), and bilirubin 1–2 mg/dL (OR: 0.68, *p* < 0.001). The model demonstrated moderate predictive accuracy (c‐statistic = 0.66). Refer to Table 3.

**TABLE 3:** Significant variables in multivariate analysis.

Variable	Odds ratio	*p*	95% CI
*Blood group*			
A	1.36	< 0.001	1.18–1.56
B	1.38	0.001	1.14–1.67
AB	2.38	< 0.001	1.60–3.54
*BMI* (*kg/m* ^*2*^)			
≤ 18	0.74	0.001	0.62–0.88
*Weight* (*kg*)			
> 40 and ≤ 50	0.69	0.036	0.48–0.98
*Race/ethnicity*			
Pacific Islander	2.95	0.05	1–8.70
African American	1.28	0.005	1.08–1.52
*Insurance type*			
Public	1.17	0.01	1.03–1.32
*Clinical parameters*			
Ascites	1.21	0.004	1.06–1.38
*Bilirubin* (*mg/dL*)			
> 1.0 and ≤ 2.0	0.68	< 0.001	0.56–0.83
*Sodium* (*mEq/L*)			
≤ 130	1.69	0.01	1.13–2.53
> 150	0.38	0.002	0.20–0.69
*Diagnosis*			
Acute hepatic necrosis	0.53	< 0.001	0.43–0.67
Biliary atresia	1.22	0.026	1.02–1.45
Metabolic disorder	2.25	< 0.001	1.79–2.84
Malignancy	2.86	< 0.001	2.15–3.79
*Center volume*			
Medium tertile	2.92	0.001	1.52–5.59
High tertile	4.11	< 0.001	2.16–7.83
*Center TV rate*			
Medium TV utilization	0.376	0.021	0.16–0.86

*Note:* The odds ratio, *p*‐value, and 95% confidence interval are listed along with the reference variable.

As outlined in the Statistical Analysis section, a higher odds ratio indicates a higher probability of transplant within 1 year of listing, and conversely, a lower odds ratio indicates a lower probability of transplant.

To evaluate the impact of graft type on transplant timing, we performed a secondary analysis which showed that recipients of Technical Variant (TV) grafts had significantly higher odds of being transplanted within 1 year compared to those waiting for a whole organ (OR 10.8, *p* < 0.001).

### 3.3 Predictive Model

**FIGURE 1:**
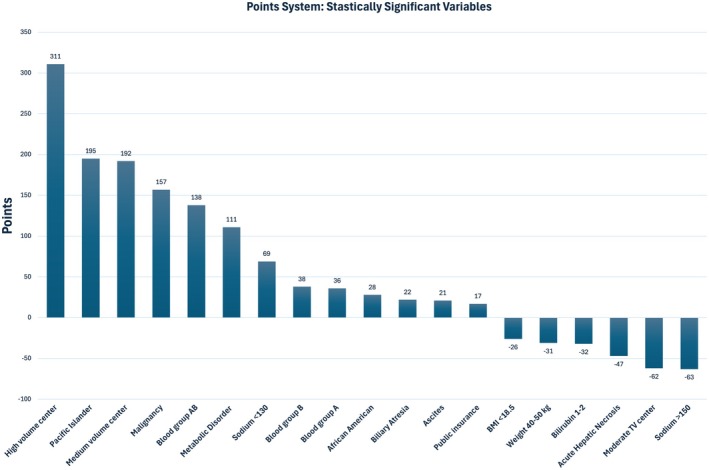
Point value assigned to each statistically significant variable.

**FIGURE 2:**
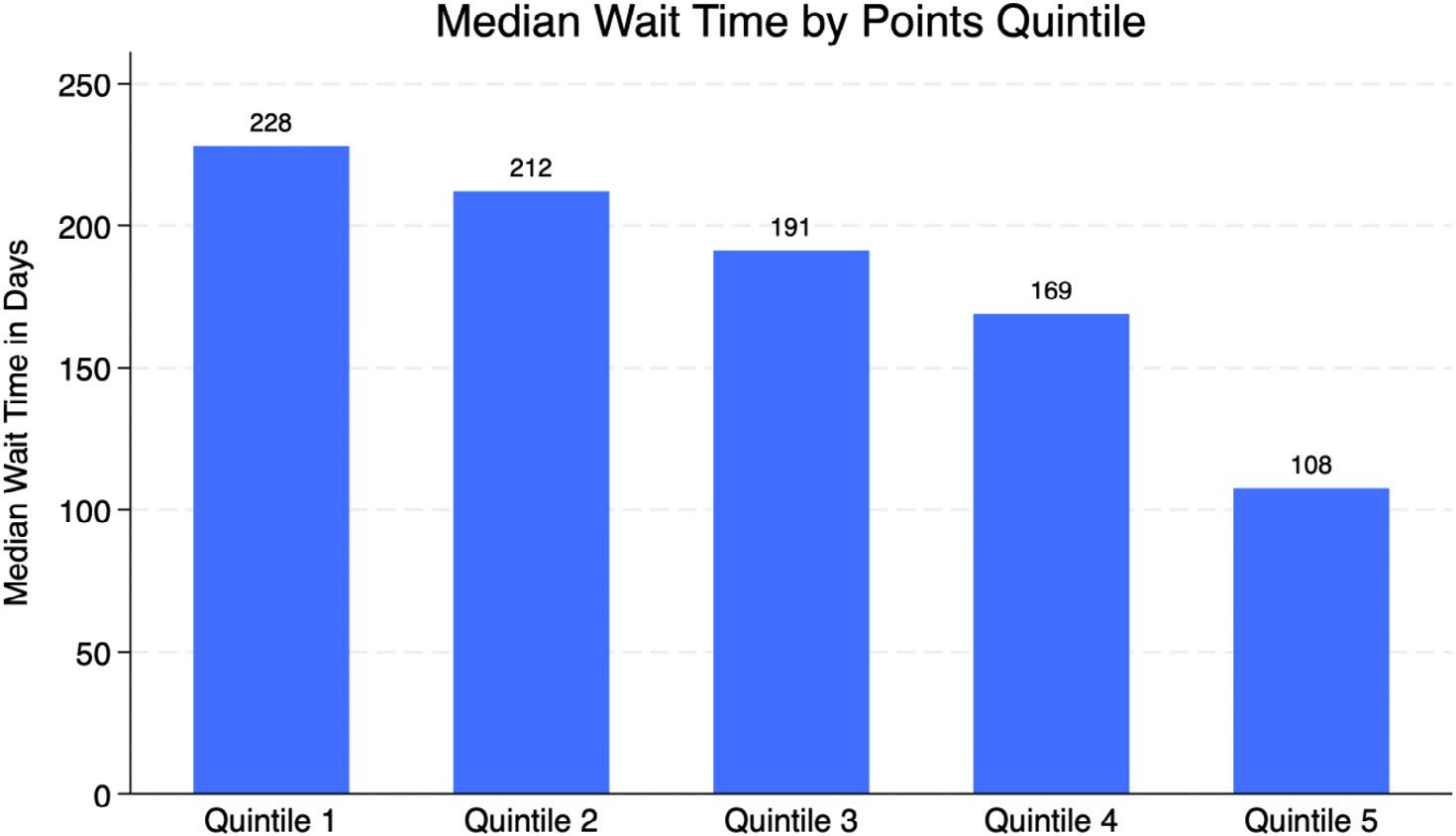
Points scores were stratified into quintiles and graphed against the median wait time in days. As quartile increases, wait time decreases. The points for each quintile are as follows: Quintile 1: ≤ 275, Quintile 2: 275–324, Quintile 3: 324–360, Quintile 4: 360–423, Quintile 5: > 423.

**FIGURE 3:**
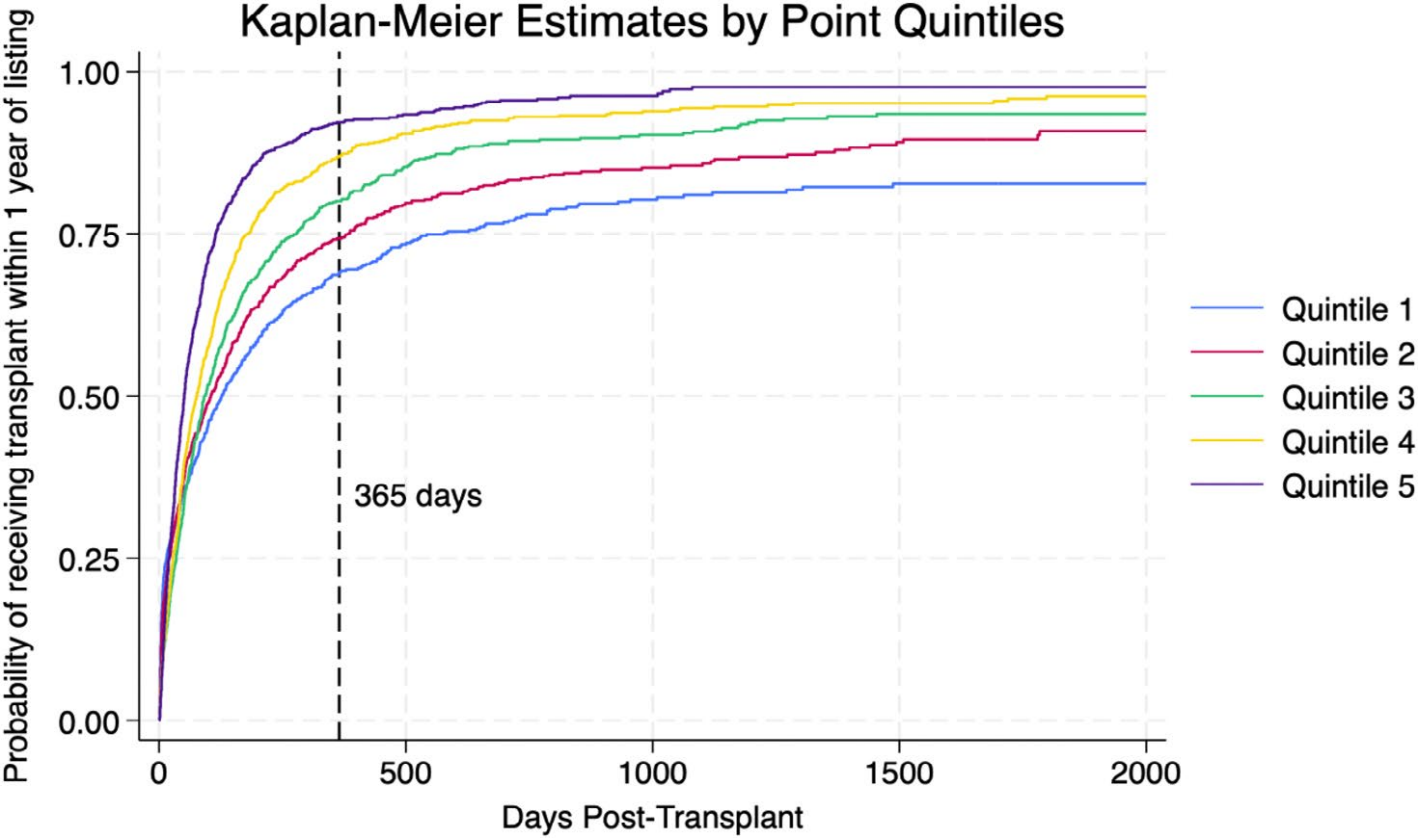
Kaplan–Meier failure estimates displaying the likelihood of transplant for each points system quintile. A reference line for 1 year post‐listing is shown. Higher quintiles consistently show greater chances of transplantation as days post‐transplant increase.

**FIGURE 4:**
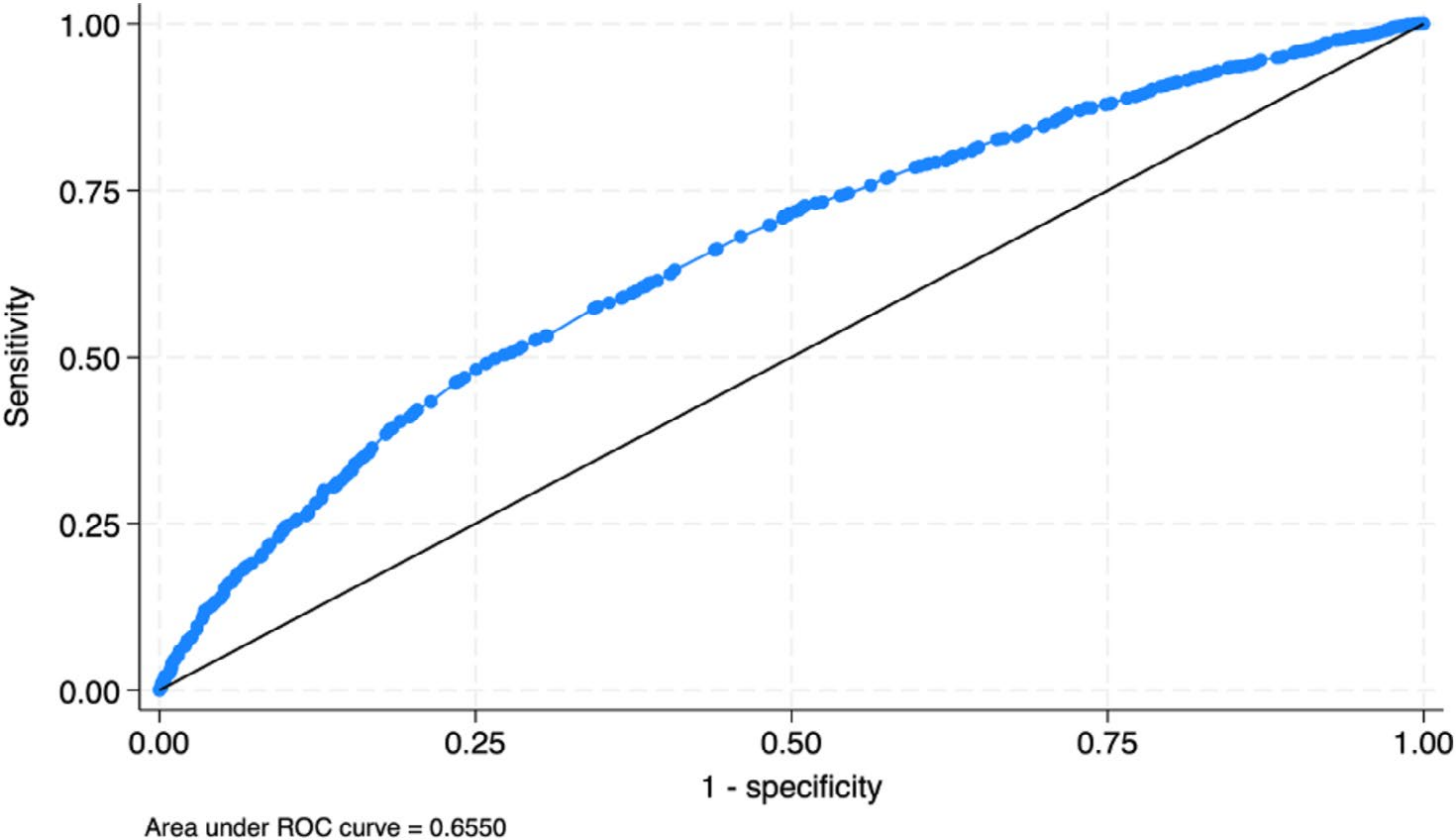
ROC curve for points system. Area under ROC is 0.655.

A points system was created using the variables found to be statistically significant in the multivariate analysis. Following the equation outlined in the methods section, points were assigned to each significant variable found in the multivariate analysis. Figure 1 depicts the point distribution of each significant variable. Candidates in the lowest quintile of the points system had an average wait time of ~228 days, while candidates in the highest quintile had an average wait time of ~108 days (Figure 2). The Kaplan–Meier curve (Figure 3) demonstrates the long‐term differences in transplant probabilities for the quintiles. An ROC analysis of the curve was conducted which yielded a c‐statistic of 0.655 (Figure 4).

## 4 Discussion

This study created a predictive points system by incorporating the 19 variables found to be statistically significant in the univariate and multivariate regression analyses. This model demonstrated strong predictive performance with a c‐statistic of 0.655 (CI: 0.639–0.671). Several variables, notably high volume transplant centers and malignancy, were associated with decreased wait times, while other variables such as adolescence and elevated sodium were associated with increased wait times.

In a previous analysis, Luo et al.'s model analyzed 6471 pediatric liver transplant candidates from 2006 to 2017 to predict timing of transplant. They analyzed demographic variables, MELD/PELD scores, region, blood type, and diagnosis to create a user‐friendly tool [13]. They found that certain variables, such as age 10–18, decreased chances of transplant, while others such as non‐O blood group increased chances. In addition to Luo et al.'s variables, our study also incorporates factors such as insurance type, dialysis status, and numerous lab values. Many variables not considered in Luo's study were found to be statistically significant. Notably, sodium and bilirubin showed strong associations. Furthermore, the importance of transplant centers, from the volume of cases to TV utilization, has a large impact on waitlist characteristics, which our study incorporates into the points system [9]. Although the type of graft received is unknown at time of listing, our transplant center characteristics was able to capture these variables in our analysis.

Our score system includes variables found to be significantly associated with increased wait times in the literature. Acute hepatic necrosis (AHN) and biliary atresia were associated with longer wait times. AHN with acute liver failure (ALF) shows shorter wait times; however, AHN with acute‐on‐chronic liver failure (ACLF) shows longer wait times. This suggests that ACLF complicates transplants in patients with AHN [14, 15]. Another variable associated with longer wait times was low sodium. The role of hyponatremia in disease acuity is well‐established; in 2016, the MELD score was updated to include serum sodium as a factor, with hyponatremic patients receiving a boost in their MELD score [16]. While the MELD update prioritizes hyponatremia to reflect acuity, our findings suggest that hypernatremic patients, who may be acutely ill, may not receive a similar allocation advantage, resulting in prolonged wait times. In the univariate analysis, centers with no TV utilization and moderate TV utilization showed a statistically significant increase in wait times compared to centers with high TV utilization times. This aligns with current literature that states that TV transplants, as well as live donor transplants, are a viable alternative with improved mortality and transplant times, especially in high volume centers [17]. Our data further illustrates this concentration of expertise, as centers in the highest volume tertile performed a median of 124 transplants and accounted for over 80% of the total study volume (Table S1). Prolonged wait times for certain candidates, such as in infants, may thus be due to center‐level challenges in utilizing split‐liver or living‐donor grafts.

Several variables were found to be associated with shorter waitlist times, for example blood group AB. Being the universal recipient, it is logical that this blood group has an easier time finding a compatible allograft organ. This is consistent with current literature which also indicates that group AB has the fastest transplant rates [13, 18]. Metabolic disorder and malignancy were found to have shorter wait times. The acuity of these conditions is well‐established in literature as these diseases tend to carry the risk of deterioration, leading to transplant priority [19, 20]. The Pacific Islander race was highly associated with decreased wait times. This is a relatively understudied demographic in the US and no literature directly studied this association, making this finding particularly interesting. Surprisingly, MELD/PELD scores were not statistically correlated with waitlist times. Table 1 shows that increasing MELD/PELD scores are associated with shorter wait times; however, they did not meet the threshold of *p* < 0.05. The reason why these scores, created to stratify candidates for transplant candidates, could be explained by the extensive use of “exception points”, which are points artificially added to a candidate's MELD/PELD score after their listing, accounting for conditions ranging from hepatoblastoma to failure to thrive. Though ~58% of candidates received these exception points, these candidates faced greater risk of pretransplant mortality, highlighting the danger of non‐standardized exceptions to the MELD/PELD score [21].

In 2020 UNOS replaced the previous regional system with an acuity circles (AC) model which allocates liver transplants based on distance from the donor hospital, not based on region. This change, along with Mazariegos et al. emphasizing that UNOS region alone is not responsible for changes in waitlist times, led to the decision to exclude UNOS/OPTN regions from the study, instead focusing on transplant‐center characteristics [9]. As above, centers with low technical graft utilization had longer WL times. Also analyzed were center transplant volumes, shown by the number of transplants in the 10‐year period. Compared to low volume centers, moderate and high‐volume centers were associated with significant decreases in waitlist times. This highlights the claim that waitlist outcomes and times extend beyond UNOS regions, and in fact is due to transplant center‐level characteristics. Candidates at lower volume centers, defined as performing < 5 transplants per year, had a significantly lower probability of receiving transplant compared to higher volume centers [22]. While the specific graft type is an outcome known only at the time of surgery, our secondary analysis confirms that TV grafts had significantly higher odds of being transplanted within 1 year compared to those waiting for a whole organ (OR 10.8, *p* < 0.001). Our data supports these institutional differences, showing that nearly half of the included programs (*n* = 39) performed no TV grafts, while high‐utilization centers achieved a median TV rate of 13.80% (Table S2). Families of children with low predicted scores can be identified early for referral to high‐volume centers where split‐liver or living‐donor options are more frequently utilized.

Building upon the findings of this study, future research should focus on the external validation of the predictive model in real‐world clinical settings. Applying the model across diverse transplant centers will help assess its generalizability and refine its predictive accuracy. Additionally, incorporating dynamic data collected post‐listing, such as changes in clinical status, laboratory values, and emergent comorbidities, could enhance the model's responsiveness and predictive capability over time. Further research is warranted to explore psychosocial and socioeconomic factors influencing waitlist times. Variables such as family support, proximity to transplant centers, and access to healthcare resources are not currently captured in the OPTN database but may significantly impact outcomes. While our model reflects the US OPTN policies, the strong influence of center volume and expertise contrasts with many European systems where mandatory splitting and living donation have already minimized waitlist delays. Our points system provides a data‐driven baseline for advocating for similar US reforms, such as standardized technical variant use, to reduce waitlist times and mortality.

As a retrospective analysis, this study is subject to inherent biases in data input, sometimes leading to missing data. While multiple rounds of imputation were done on incomplete data, the possibility of confounding remains. Furthermore, this study focuses on data points recorded at the time of listing, which does not reflect any changes post‐listing that may influence waitlist outcomes. Another limitation is the fact that living donors were excluded from this study because they undergo different listing procedures than deceased donors. This was done to preserve the predictability of the system, but we recognize that this leaves out a portion of pediatric liver donations. A primary limitation of this study is its reliance on the OPTN/UNOS database, which restricts the generalizability of the points system to countries with different allocation frameworks, particularly those with mandatory splitting or higher living‐donor utilization. Additionally, while we utilized waitlist time as our primary endpoint, we acknowledge that waitlist mortality is the ultimate clinical concern. Future research should apply this model's framework to specifically identify predictors of mortality to further refine its utility in the highest‐risk pediatric populations.

## 5 Conclusion

This study created a points system to predict waitlist times for pediatric liver transplants. This model was created after identifying 19 variables that significantly influenced wait times within 1 year of being placed on the waitlist, yielding a c‐statistic of 0.655. This predictive model is intended for supplemental use by clinicians to guide their management of pediatric liver transplants, as well as a tool that physicians can use to pursue TV evaluation or referral when a whole‐organ match is statistically unlikely. Patients and their families will also benefit by receiving new data points that will hopefully reduce the uncertainty and anxiety that surrounds the transplant process. Ultimately, continued research and collaboration among healthcare providers, policymakers, and researchers are essential to translate these findings into meaningful improvements in patient outcomes. Enhancing the predictive model will contribute to a more efficient, equitable, and compassionate pediatric liver transplant system, ultimately improving survival rates and quality of life for affected children and their families.

## Funding

The authors have nothing to report.

## Conflicts of Interest

The authors declare no conflicts of interest.

## Supporting information


**Table S1:** Distribution of pediatric liver transplant activity across transplant centers categorized by volume tertiles.

**Table S2:** Summary of technical variant (TV) graft utilization rates and center‐level distribution.

## Data Availability

The data that support the findings of this study are available from the corresponding author upon reasonable request.
